# Accuracy of StepWatch™ and ActiGraph Accelerometers for Measuring Steps Taken among Persons with Multiple Sclerosis

**DOI:** 10.1371/journal.pone.0093511

**Published:** 2014-04-08

**Authors:** Brian M. Sandroff, Robert W. Motl, Lara A. Pilutti, Yvonne C. Learmonth, Ipek Ensari, Deirdre Dlugonski, Rachel E. Klaren, Swathi Balantrapu, Barry J. Riskin

**Affiliations:** Department of Kinesiology and Community Health, University of Illinois at Urbana-Champaign, Champaign, Illinois, United States of America; Charité University Medicine Berlin, Germany

## Abstract

**Introduction:**

There has been increased interest in the objective monitoring of free-living walking behavior using accelerometers in clinical research involving persons with multiple sclerosis (MS). The current investigation examined and compared the accuracy of the StepWatch activity monitor and ActiGraph model GT3X+ accelerometer for capturing steps taken during various speeds of prolonged, over-ground ambulation in persons with MS who had mild, moderate, and severe disability.

**Methods:**

Sixty-three persons with MS underwent a neurological examination for generation of an EDSS score and undertook two trials of walking on the GAITRite electronic walkway. Participants were fitted with accelerometers, and undertook three modified six-minute walk (6MW) tests that were interspersed with 10–15 minutes of rest. The first 6MW was undertaken at a comfortable walking speed (CWS), and the two remaining 6MW tests were undertaken above (faster walking speed; FWS) or below (slower walking speed; SWS) the participant's CWS. The actual number of steps taken was counted through direct observation using hand-tally counters.

**Results:**

The StepWatch activity monitor (99.8%–99.9%) and ActiGraph model GT3X+ accelerometer (95.6%–97.4%) both demonstrated highly accurate measurement of steps taken under CWS and FWS conditions. The StepWatch had better accuracy (99.0%) than the ActiGraph (95.5%) in the overall sample under the SWS condition, and this was particularly apparent in those with severe disability (StepWatch: 95.7%; ActiGraph: 87.3%). The inaccuracy in measurement for the ActiGraph was associated with alterations of gait (e.g., slower gait velocity, shorter step length, wider base of support).

**Conclusions:**

This research will help inform the choice of accelerometer to be adopted in clinical trials of MS wherein the monitoring of free-living walking behavior is of particular value.

## Introduction

There has been increasing interest in approaches for objectively monitoring the status of persons with multiple sclerosis (MS) under real-world conditions [1]–[3]. This interest has highlighted the potential value of objectively monitoring free-living walking behavior using accelerometers in clinical research involving persons with neurological diseases including MS [1]–[3]. Accelerometers are motion sensors worn on the body (e.g., around the waist or ankle) during the waking hours of the day and over a representative sampling period (e.g., seven days). The devices capture and record the total amount of walking undertaken in free-living conditions based on the metric of steps taken per day (steps/day). The number of steps/day reflects a straight-forward metric of the overall amount of walking undertaken during one's everyday life (i.e., free-living walking behavior) [3], [4] and is a marker of health and disability status in MS [5]–[7]. There are some devices that provide measures of walking speed (e.g., ActiBelt; [8]), but this metric is not easily interpretable by researchers, clinicians, and patients. There further are guidelines for easily interpreting steps/day [9], but this is not the case for walking speed. Acceleration counts represent another possible metric for quantifying ambulation, but such a metric does not have much meaning for clinicians and patients.

Importantly, the successful application of accelerometers for measuring free-living walking behavior depends on selecting a device with acceptable accuracy for capturing steps taken during ambulation in persons with MS. Such an assessment of accuracy should be established under controlled conditions (i.e., laboratory settings) before investing considerable time and resources into an investigation under more ecologically valid conditions. The assessment of accuracy is important as the gait patterns of persons with neurological diseases such as MS can present challenges for devices that measure steps or strides during ambulation [10]. To date, there are two published investigations regarding the accuracy of accelerometers for capturing steps taken during ambulation in persons with MS [4], [11]. One study reported that an ActiGraph accelerometer (model 7164) worn around the waist accurately measured steps taken while walking on a treadmill for 6 minutes at 2.5 (99.8% accuracy rate) and 3.0 (99.7% accuracy rate) mph in 24 adults with mild MS; there was minimal inaccuracy (4.1% error rate or 95.9% accuracy rate) while walking at 2.0 mph [4]. Another study reported that the StepWatch Activity Monitor worn around the ankle accurately measured strides (98.1% accuracy rate) compared with a GaitMat II in 20 persons with Parkinson's disease and MS during 3.87 meters of self-paced, over-ground walking [11]. There have been no published reports of direct comparisons of accelerometer accuracy for capturing steps taken during prolonged periods of over-ground walking across the spectrum of MS disability status.

One approach for measuring the accuracy of accelerometers for detecting steps during over-ground walking involves the systematic manipulation of speed during consecutive 6-minute walk (6MW) tests. The 6MW has been considered a proxy of community ambulation in MS [7], [12] and persons with Expanded Disability Status Scale (EDSS) scores between 0 and 6.5 can perform repeated 6MW tests [13]. Indeed, previous research has delivered three, over-ground 6MW tests to successfully manipulate speed and examine the O_2_ cost of walking in persons with MS [14], [15]. In those studies, one 6MW was performed at the participant's comfortable walking speed (CWS), whereas the other two 6MW tests were undertaken above (faster walking speed; FWS) and below (slower walking speed; SWS) the participant's CWS [14], [15]. Such an approach might be advantageous for an examination and comparison of accelerometer accuracy across different conditions of ambulation and levels of disability.

The current investigation examined and compared the accuracy of the StepWatch activity monitor and ActiGraph model GT3X+ accelerometer for capturing steps taken during various speeds of prolonged, over-ground ambulation in a sample of persons with MS who had mild, moderate, and severe disability. This extends the ecological validity of accelerometer accuracy trials from a treadmill walking protocol (e.g., [4]) to an over-ground walking protocol, prior to examining accuracy under free-living conditions. This is an intermediary condition between the treadmill and real world, and reflects the progression of confirmation before testing under real-world conditions. Importantly, this study included commercially-available ActiGraph and StepWatch devices, as these are the most commonly used accelerometers for measuring ambulation in healthy and disease populations, including MS [10].

## Methods

### Participants

Prospective participants residing in the local community (i.e., within 60 minutes of our laboratory) were contacted by a flyer that was distributed amongst participants from previously conducted studies in our laboratory. Prospective participants were further recruited via telephone and e-mail messages along with referrals from a local neurologist. Those who expressed interest in the study underwent a screening for inclusion criteria that included a neurologist-confirmed diagnosis of MS, relapse free during the previous 30 days [16], ambulatory with or without assistance, age between 18 and 65 years, and absence of risk-factors for undertaking strenuous physical activity (e.g., cardiovascular diseases, diabetes, hyperlipidemia, and hypertension). As we were interested in forming groups of mild, moderate, and severe disability, prospective participants underwent a self-reported EDSS [17] over the telephone. The resultant score was not used in data analyses, but was used as a preliminary indicator of disability status for the purpose of recruiting disability subgroups of relatively equal size. As an inclusion criterion involved being ambulatory, we recruited persons with MS who had a maximum EDSS score of 6.5 (i.e., constant bilateral assistance). An EDSS score of 7.0 (i.e., the next level of disability) reflects the inability to walk with an assistive device for more than 5 meters, and reflects being regularly wheelchair-bound. We felt that it would not be possible to examine and compare accelerometer accuracy for detecting steps taken by including persons with this severe a level of disability. We contacted 148 persons with MS, and 61 were uninterested in participating. The resulting 87 persons underwent screening, and 15 qualified for the study, but were unable to travel to our laboratory, and 9 persons were disqualified based on the presence of risk-factors for undertaking physical activity. The final sample consisted of 63 persons with MS.

### Accelerometers

Steps taken during the 6MW tests were measured by an ActiGraph model GT3X+ accelerometer (Health One Technology, Fort Walton Beach, FL) and a StepWatch Activity Monitor (Orthocare Innovations, Mountlake Terrace, WA). The ActiGraph model GT3X+ contains a solid state, digital accelerometer that generates an electrical signal proportional to the force acting on it along three axes. The acceleration signal is sampled by a 12-bit analog-to-digital converter and stored in a raw format in the units of gravity (G's). The raw activity data are post-processed in ActiLife 6 software and are expressed as step counts; this study only included step counts for consistency with the StepWatch. The StepWatch activity monitor is a microprocessor-controlled, two-dimensional accelerometer that measures step counts and step rates per unit time. The threshold for acceleration is stored in the unit's memory in G's and is calibrated by the manufacturer (Orthocare Innovations, Mountlake Terrace, WA); the threshold for all units in the current study was 1.08 G's.

The ActiGraph was worn on an elastic belt around the waist and above the right hip, whereas the StepWatch was worn on an elastic strap around the ankle above the right lateral malleolus, as per manufacturer recommendations. The epoch (i.e, sampling window) for both the ActiGraph and StepWatch was 3 seconds (i.e., this is the shortest epoch available for the StepWatch). The short epochs were chosen for flexibility in data processing and precise linking with the exact beginning and end of the 6MW tests. The data from both accelerometers were imported into Microsoft Excel for processing. We used five separate ActiGraph model GT3X+ accelerometers and five separate StepWatch activity monitors in the study, and counterbalanced the application of each of these units, such that each individual accelerometer was used at least 3 times within each disability status group. We further checked the accuracy of all accelerometers for capturing 1000 steps while walking on a treadmill at 3.0 mph using laboratory personnel before beginning the research and upon its completion; this ensured proper functioning of the devices before and after the research protocol. The ActiGraph model GT3X+ was initialized using the low-frequency extension feature, in order to increase the accelerometer's sensitivity for capturing low frequency accelerations (i.e., slow walking). We further set the StepWatch activity monitor to flash for the first 50 steps taken to ensure functionality prior to the first 6MW. To do this, once properly fitted with the StepWatch, we instructed the participant to take 10 steps prior to the first 6MW administration to verify that the unit was indeed flashing and thus measuring steps taken.

### Disability status

All participants underwent a neurological exam by a Neurostatus certified examiner who generated EDSS scores [18] for describing the sample and stratifying persons into three groups based on mild (EDSS of 0–3.5; n = 20), moderate (EDSS of 4.0–5.5; n = 24), and severe (EDSS of 6.0–6.5; n = 19) disability status.

### Walking/gait outcomes

Walking and gait measures were included for identifying possible sources of error in accelerometer output. The MSWS-12 is a 12-item, patient-rated measure of the impact of MS on walking-related activities (including walking, running, standing, climbing stairs) [19]. The items are rated on a 5-point scale of 1 (*Not at all*) to 5 (*Extremely*), and the items represent limitations of walking during the past 2 weeks. The MSWS-12 is scored by summing the item scores, subtracting 12, dividing the difference by 48, and then multiplying the result by 100. The final score ranges between 0 and 100 and higher scores are indicative of worse walking. The MSWS-12 has good evidence for its internal consistency, test-retest reliability, longitudinal invariance, and validity of scores as a measure of walking mobility in MS [20].

Participants completed two trials of walking on a 16-foot GAITRite (CIR systems, Inc) electronic walkway at a comfortable, self-selected pace for measuring gait outcomes. We recorded the functional ambulation profile (FAP) score, velocity (cm/sec), cadence (steps/min), step length (cm), step time (sec), base of support (cm), and double support (%). We averaged the recorded values per variable across both trials for improved reliability.

### Ethics Statement

The procedure was approved by the University of Illinois at Urbana-Champaign Institutional Review Board and all participants provided written informed consent before beginning the study.

### Protocol

Participants completed a demographic questionnaire and the MSWS-12, followed by a neurological examination for generation of an EDSS score. Participants then undertook two trials of walking on the GAITRite electronic walkway. One researcher then guided each participant through the course for the 6MW tests as a familiarization protocol. The course was located in an accessible, rectangular hallway that was clear of obstructions and foot traffic. The StepWatch and ActiGraph accelerometers were then properly positioned on each participant. Participants completed the three 6MW tests that were interspersed with 10–15 minutes of rest. The first 6MW test involved the participant's CWS, and the two remaining 6MW tests were undertaken above (FWS; +0.5 mph of CWS) or below (SWS; −0.5 mph of CWS) the participant's CWS; the order of the FWS and SWS 6MW tests were counterbalanced. During the CWS 6MW test, one researcher followed the participant and recorded the total distance walked using a measuring wheel (Keson MP301, Aurora, IL), which was further outfitted with a pre-calibrated bicycle computer (Cateye Velo5, Osaka, Japan). Based on the total distance walked and time (i.e., 6 minutes), average speed for CWS was calculated in miles per hour (mph) using Microsoft Excel. The manipulation of walking speed in the 2^nd^ and 3^rd^ 6MW tests was accomplished by having the participant follow a researcher who controlled the walking pace using the Keson measuring wheel outfitted with the bicycle computer; we recorded total distance and using time, computed average speed as a manipulation check [14], [15]. For all three 6MW tests, the actual number of steps taken was further counted by a laboratory research assistant through direct observation using a hand-tally counter. We recognize that having the participants follow a researcher during FWS and SWS conditions involves a dual-task of walking and the participant cognitively adapting the pace of his/her walking to that of the researcher. This might bias 6MW distance and could be a major confound for comparing 6MW distance values herein with other studies. Nevertheless, this protocol was necessary for manipulating the speed of over-ground walking in order to test the accuracy of the accelerometers under different walking conditions. The current 6MW distance values are not comparable to those from studies that use a standard 6MW protocol. Participants received $25 remuneration upon completion of the study.

### Data Analysis

The data analyses were performed using IBM SPSS Statistics, Version 21 (SPSS Inc., Chicago, IL) and the data are available upon written request and approval by the University of Illinois. Descriptive data are presented as mean scores (standard deviation; *SD*), unless otherwise noted. Step counts measured by the accelerometers are expressed as a percentage of the actual number of steps taken measured by direct observation during each of the 6MW tests (i.e., device accuracy). That was the primary dependent variable and values less than 100% reflect underestimation of step counts by the accelerometer, whereas values greater than 100% reflect overestimation of step counts by the accelerometer. We first conducted a 3 (Condition: CWS, FWS, and SWS)×3 (Group: mild, moderate, and severe) mixed-model ANOVA on the outcome of walking speed. Condition was a within-subjects factor, and group was a between-subjects factor. This analysis served as a manipulation check of our protocol across the three walking conditions and disability levels. We then conducted a 2 (Device: StepWatch and ActiGraph)×3 (Speed: CWS, FWS, SWS)×3 (Group: mild, moderate, and severe) mixed-model ANOVA on the outcome of device accuracy. Device and condition were within-subjects factors, and group was a between-subjects factor. This analysis examined differences in the accuracy of the devices overall and as a function of speed and disability status. We did not correct for alpha because our *a priori* data analysis plan involved examining accelerometer accuracy in two devices among three disability groups. Finally, we were interested in walking and gait outcomes (e.g., MSWS-12, GAITRite variables, and assistive device use during the 6MW tests) as potential influences on any inaccuracy in measurement. Thus, we examined the correlations between walking/gait outcomes and accuracy of measurement of the device/speed combination that measured the lowest percentage of actual steps taken (i.e., the greatest inaccuracy in measurement) using bivariate, Pearson correlation analyses and one-way ANOVA.

## Results

### Sample Characteristics

Demographic, clinical, and walking/gait characteristics of the sample based on disability status are presented in Table 1. Briefly, we enrolled 63 persons with a definite diagnosis of MS. The sample was largely female (*n* = 48 or 76%) with an average age of 50.7 (9.2) years. Regarding clinical course of MS, 50 (79%) of the participants had relapsing-remitting MS and 12 (19%) had progressive MS; 1 participant had an unknown clinical course. The mean duration of MS (i.e., time since diagnosis) was 12.8 (8.5) years, and median EDSS score was 4.0 with a range of scores between 1.0 (i.e., minimal disability) and 6.5 (i.e., constant use of bilateral assistance during walking) (IQR = 2.5). Overall, most participants (i.e., 40/63; 64%) did not require the use of an assistive device during the 6MW tests; 13 participants (21%) used a cane, and 10 (16%) used a rollator. The current sample had walking/gait characteristics that were similar to other samples of persons with mild, moderate, and severe disability [21].

**Table 1:** Demographic, Clinical, and Walking/Gait Characteristics of 63 persons with mild, moderate, and severe MS.

Variable	Overall (n = 63)	Mild (n = 20)	Moderate (n = 24)	Severe (n = 19)
Age (years)	50.68 (9.22)	48.25 (10.68)	52.71 (7.97)	50.68 (8.91)
Sex (n, % female)	48/63 (76.2%)	14/20 (70.0%)	19/24 (79.2%)	15/19 (78.9%)
MS Duration (years)	12.83 (8.50)	10.80 (7.71)	14.58 (7.39)	12.74 (10.38)
MS Type (n,% RRMS)	50/63 (79.4%)	20/20 (100%)	18/23 (75.0%)	12/19 (63.2%)
EDSS (median, range)	4.0 (1.0–6.5)	3.0 (1.0–3.5)	4.0 (4.0–5.0)	6.0 (6.0–6.5)
6MW AD-None (n, %)	40/63 (63.5%)	19/20 (95.0%)	20/24 (83.3%)	1/19 (5.3%)
6MW AD-Cane (n, %)	13/63 (20.6%)	1/20 (5.0%)	4/24 (16.7%)	8/19 (47.4%)
6MW AD-Rollator (n, %)	10/63 (15.9%)	0/20 (0.0%)	0/24 (0.0%)	10/19 (52.6%)
FAP	87.32 (15.4)	96.42 (3.2)	92.42 (6.4)	71.79 (19.1)
Gait Velocity (cm/sec)	100.43 (30.1)	125.05 (18.6)	106.45 (21.2)	68.21 (19.1)
Cadence (steps/min)	101.19 (16.0)	110.25 (8.5)	106.99 (11.0)	84.82 (15.2)
Step Length (cm)	58.72 (11.3)	68.10 (8.2)	59.53 (8.4)	48.33 (8.2)
Step Time (sec)	0.64 (0.18)	0.55 (0.04)	0.57 (0.06)	0.81 (.23)
Base of Support (cm)	12.06 (4.2)	11.08 (3.0)	11.38 (2.9)	13.89 (6.0)
Double Support (%)	33.94 (14.5)	27.34 (4.1)	31.37 (4.4)	43.80 (22.7)
MSWS-12	40.57 (29.2)	11.98 (12.4)	41.58 (20.6)	72.79 (16.4)

Note: Data are presented as mean (SD) unless otherwise noted; 1 participant with moderate disability did not provide MS type; RRMS = Relapsing-remitting multiple sclerosis; EDSS = Expanded Disability Status Scale; 6MW = 6-minute walk; AD = Assistive Device; FAP = Functional ambulation profile; MSWS-12 = Multiple Sclerosis Walking Scale-12.

### Speed Manipulation Check

All 63 participants completed the three 6MW tests without stopping or obvious difficulty. Average speeds for the CWS, FWS, and SWS 6MW tests in the overall sample and by subsamples with mild, moderate, and severe MS are reported in Table 2. The ANOVA indicated a significant main effect for speed (*F*(2, 120) = 477.32, *p*<.001, eta-squared = 0.89) supporting our successful manipulation of speed across the 6MW tests. There further was a significant main effect for disability (F(2, 60) = 47.09, *p*<.001, eta-squared = .61) indicating that actual speed (and ultimately distance) differed between mild, moderate, and severe MS. The manipulation of speed did not differ as a function of disability status based on a non-significant speed×disability interaction (*F*(4, 120) = 1.92, *p* = .11, eta-squared = .06).

**Table 2:** Average Speed under comfortable, fast, and slow walking conditions in 63 persons with mild, moderate, and severe MS.

Condition	Disability	Average Speed (mph)
CWS	Overall	2.17 (0.63)
	Mild	2.66 (0.42)
	Moderate	2.34 (0.35)
	Severe	1.45 (0.41)
FWS	Overall	2.61 (0.71)
	Mild	3.16 (0.42)
	Moderate	2.81 (0.35)
	Severe	1.79 (0.56)
SWS	Overall	1.71 (0.61)
	Mild	2.16 (0.40)
	Moderate	1.87 (0.36)
	Severe	1.02 (0.40)

Note: Data are presented as mean (SD); CWS = Comfortable walking speed; FWS = Faster walking speed; SWS = Slower walking speed.

### Device Accuracy

Table 3 presents actual steps taken during the 6MW tests and those measured by the ActiGraph and StepWatch in the overall sample and per disability group. Table 3 further expresses these data as percentage of actual steps taken measured by each device. Results from the primary analysis for comparing accuracy (i.e., percentage of the actual number of steps taken) per device as a function of speed and disability status are as follows. There was not a significant device×speed×disability interaction (*F*(4, 120) = 1.14, *p* = .34, eta-squared = .04), indicating that there was not a difference in accuracy between the ActiGraph and StepWatch across speed as a function of disability. The accuracy of devices did not vary as a function of speed based on a non-significant device×speed interaction (*F*(2, 120) = 0.27, *p* = .76, eta-squared = .01). There further was a non-significant device×disability interaction (*F*(2, 60) = 2.58, *p* = .08, eta-squared = .08) indicating that accuracy of the ActiGraph and StepWatch did not significantly differ across disability groups. There was a significant speed×disability interaction (*F*(4, 120) = 2.40, *p* = .05, eta-squared = .07) indicating that there was a greater inaccuracy (i.e., smaller percentage of actual steps taken measured by the accelerometers) as a function of speed per level of disability, such that inaccuracy was greatest in those with severe disability under the SWS condition. There was a significant main effect for device (*F*(1, 60) = 5.27, *p* = .03, eta-squared = .08) indicating that, overall, the StepWatch was slightly more accurate than the ActiGraph model GT3X+ accelerometer.

**Table 3:** Actual and accelerometer-measured steps taken and accelerometer accuracy in 63 persons with mild, moderate, and severe MS.

Speed	Disability	Steps Taken			Percentage of Actual Steps Taken Measured by Accelerometers	
		Actual	ActiGraph	StepWatch	ActiGraph	StepWatch
CWS	Overall	591.0 (97.2)	575.6 (116.0)	589.6 (102.6)	97.4%	99.8%
	Mild	641.0 (54.4)	639.8 (59.0)	640.4 (58.8)	99.8%	99.9%
	Moderate	634.0 (54.4)	629.0 (62.6)	645.6 (56.4)	99.2%	101.8%
	Severe	484.8 (91.4)	440.6 (100.0)	478.4 (99.2)	90.9%	98.7%
FWS	Overall	645.8 (103.8)	617.4 (126.6)	645.2 (101.6)	95.6%	99.9%
	Mild	698.8 (58.2)	698.6 (63.6)	696.2 (58.0)	100.0%	99.6%
	Moderate	690.0 (52.4)	684.0 (58.8)	690.8 (53.8)	99.1%	100.1%
	Severe	529.0 (102.4)	480.8 (118.0)	534.0 (97.6)	90.9%	100.9%
SWS	Overall	520.6 (107.4)	497.0 (132.4)	515.6 (119.0)	95.5%	99.0%
	Mild	572.2 (64.8)	574.6 (68.0)	572.0 (68.0)	100.4%	100.0%
	Moderate	573.8 (55.8)	549.4 (81.8)	574.2 (56.6)	95.7%	100.1%
	Severe	400.0 (95.6)	349.2 (116.4)	382.6 (114.0)	87.3%	95.7%

Note: Data are presented as mean (SD); CWS = Comfortable walking speed; FWS = Faster walking speed; SWS = Slower walking speed.

We performed an additional exploratory analysis of device accuracy within groups based on “normal” walking speed. As such, *post-hoc*, we re-categorized persons into three groups based on normal walking speed recorded from the GaitRite (i.e., stratified as ‘community walkers’, ‘limited community walkers’, and ‘community walkers’; [22], [23]). Using those groups, there was a statistically significant speed×group interaction (*p* = .01) on device accuracy (i.e., percentage of actual steps taken measured by the devices) indicating that there was a greater inaccuracy (i.e., smaller percentage of actual steps taken measured by the devices) as a function of speed (i.e., CWS, FWS, SWS) per group, such that inaccuracy was greatest in those classified as ‘most limited community walkers’ under SWS conditions. There further was a significant device main effect (*p* = .02) such that, overall, the StepWatch measured a greater percentage of actual steps taken than the ActiGraph model GT3X+ accelerometer. Collectively, this pattern of results when examining groups based on normal walking speed is entirely consistent with the device accuracy results when analyzing the data based on disability status.

### Associations Among Device Accuracy and Walking/Gait Variables

We were interested in examining possible walking or gait-related influences on the accuracy in measurement between device types. This was unnecessary for the StepWatch because of its accuracy in measurement under all walking conditions and for the ActiGraph model GT3X+ under CWS and FWS conditions. Accordingly, we only report the correlations between walking/gait outcomes and the accuracy in measurement of the ActiGraph model GT3X+ accelerometer under SWS conditions (i.e., the speed and device combination with the greatest inaccuracy in measurement). Bivariate correlations indicated that device accuracy (i.e., percentage of the actual number of steps measured) was significantly associated with FAP scores (*r* = .361, *p* = .004), velocity (*r* = .351, *p* = .005), step length (*r* = .376, *p* = .003), step time (*r* = −.352, *p* = .005), base of support (*r* = −.255, *p* = .046), double support (*r* = −.356, *p* = .005), and MSWS-12 scores (*r* = −.290, *p* = .023). One-way ANOVA indicated that there were not statistically significant differences in accuracy in measurement of the ActiGraph model GT3X+ accelerometer based on the use of an assistive device (i.e., none, cane, or rollator) during the 6MW tests (*F*(2, 60) = 0.37, *p* = .69).

## Discussion

The current investigation examined the accuracy of the ActiGraph model GT3X+ accelerometer and the StepWatch activity monitor for measuring steps taken during various speeds of over-ground walking in persons with mild, moderate, and severe MS. All participants were able to complete each 6MW test without incident, including those with severe disability. This is consistent with seminal research involving the administration and tolerability of three maximal 6MW tests in persons with mild, moderate, and severe MS [13]. The current approach further was successful in the manipulation of walking speed across the three levels of disability status. The primary novel findings were that under the CWS and FWS conditions of the 6MW, both the ActiGraph model GT3X+ accelerometer (95.6%–97.4%; i.e., 4.4%–2.6% underestimation of steps) and StepWatch activity monitor (99.8%–99.9%; i.e., 0.2%–0.1% underestimation of steps) demonstrated highly accurate measurement of actual steps taken. As the CWS condition involved a self-selected pace, and presumably is the most common walking pace at which free-living ambulation occurs, the current accuracy results would support the use of either device in clinical research applications for measuring free-living steps/day in persons with MS. However, under the SWS condition of the 6MW (i.e., 0.5 mph slower than CWS), it is of note that the StepWatch measured a greater percentage of actual steps taken (99.0%) than the ActiGraph (95.5%) in the overall sample, and this was particularly apparent in those with severe disability (StepWatch: 95.7%; ActiGraph: 87.3%). This inaccuracy further was observed in persons who were classified as ‘most limited community walkers’ under SWS conditions. This slight inaccuracy in measurement for the ActiGraph might be attributed to altered spatiotemporal parameters of gait (i.e., slower velocity, shorter step length, longer step time, wider base of support, and more time spent in double support of the gait cycle) and greater perceived impact of walking impairment. Perhaps the ActiGraph device is not sensitive enough for capturing bodily displacement during slow walking speed among those with severe disability characterized by altered gait.

Importantly, previous studies of the accuracy in measurement of these motion sensors were unable to capture potential walking and gait-related sources of error, as such studies have been limited in sample size and only included persons with a narrow range of MS disability [4], [11]. One study reported that the ActiGraph model 7164 accelerometer (a precursor to the model GT3X+) accurately measured steps taken in persons with mild MS while walking at 2.0 mph (95.1%), 2.5 mph (99.8%), and 3.0 mph (99.7%) on a motor-driven treadmill, respectively [4]. The current results are consistent with those values in persons with mild MS (measurement of 100.4% of steps taken for SWS (i.e., 0.4% overestimation); 99.8% of steps taken for CWS (i.e., 0.2% underestimation); and 100.0% of steps taken for FWS), and extend results from that previous investigation by providing accuracy measurements in individuals with moderate and severe MS disability. The current accuracy results for the StepWatch are consistent with a previous study that reported 98.1% accuracy for the StepWatch during 3.87 m of comfortable walking in 9 women with moderate MS disability [11]. We report that under CWS conditions for a longer duration of time (i.e., 6 minutes), the StepWatch measured 101.8% of steps taken (i.e., 1.8% overestimation) in persons with moderate MS disability, and similarly accurate measurement of steps taken in persons with mild (99.9%; i.e., 0.1% underestimation) and severe (98.7%; i.e., 1.3% underestimation) disability. Collectively, this has potential implications for the interpretation of results from previous investigations using the ActiGraph GT3X+ as a measure of steps/day, such that this accelerometer might be underestimating steps during slow walking speeds, particularly in individuals with severe disability who have altered gait kinematics. For example, one recent study reported that persons with MS take an average of 5,826 steps/day based on step counts from ActiGraph accelerometers [5]. Those results should be interpreted with caution, such that persons with MS might actually be participating in slightly more physical activity, based on the potential underestimation of steps/day by the ActiGraph model GT3X+.

The current results have potential implications for future research. Importantly, the current over-ground walking protocol was conducted under highly-controlled laboratory conditions and represents an intermediary condition between the treadmill and real world (i.e., this reflects the progression of confirmation before testing under real-world conditions). The overall accuracy in measurement of the ActiGraph and StepWatch under CWS and FWS conditions supports the inclusion of either unit in future clinical research in persons with MS examining steps/day as a free-living measure of ambulation. The StepWatch did demonstrate slightly better accuracy than the ActiGraph overall, although this difference in accuracy was minimal across different speeds, based on a non-significant speed×device interaction. Though these differences were negligible in persons with mild and moderate MS disability, and during CWS and FWS conditions, there was a slight reduction in accuracy for both motion sensors during SWS, particularly among persons with severe MS disability. There was a greater discrepancy in accuracy during SWS across devices in persons with severe disability such that the StepWatch still measured 95.7% of steps taken (i.e., 4.3% underestimation), whereas the ActiGraph accurately measured 87.3% of steps taken (i.e., 12.7% underestimation), although this interaction was not statistically significant. This discrepancy would seemingly favor the StepWatch over the ActiGraph GT3X+ for measuring steps in clinical samples with severe disability, though it is unclear if this difference is sufficient to overwhelmingly favor the StepWatch over the ActiGraph GT3X+.

Though the StepWatch demonstrated greater accuracy than the ActiGraph model GT3X+ overall, it comes at somewhat of a cost, possibly limiting the utility of this device. The StepWatch activity monitor only measures step counts and step rates as measures of ambulatory physical activity; this device cannot express a quantification of the body's positive and negative accelerations (i.e., activity counts) to provide information about the intensity of short bouts of physical activity, whereas the ActiGraph model GT3X+ is capable of quantifying such accelerations as activity counts. This is important for understanding whether or not persons with MS are meeting public health guidelines for physical activity (i.e., 150 minutes per week of moderate-to-vigorous physical activity). The StepWatch is not as commonly used as ActiGraph accelerometers for measuring ambulatory physical activity in persons with MS and the GT3X+ is the newest commercially-available model, such that making comparisons across different samples can be difficult [2]. Finally, although highly accurate, the StepWatch is quite expensive compared with the ActiGraph model GT3X+, and might be a less feasible option for large-scale clinical research endeavors.

There were many strengths of the current study including the inclusion of over-ground walking at 3 different speeds and a relatively large sample of persons with varying MS disability, but it is not without limitations. The two primary limitations of this study were that we compared the accuracy of only 2 devices at only 3 different speeds. ActiGraph and StepWatch accelerometers represent the two most commonly used accelerometers for measuring ambulation in healthy and disease populations, including MS [10]. Further, it was practical to compare the accuracy of these units as each is affixed to a different location on the body during ambulation; there is likely a limit to the number of accelerometers that can be simultaneously positioned on the same bodily location without influencing accuracy. Future research might examine the accuracy of other types of motion sensors (e.g., Actical, TriTac accelerometers) for measuring steps under a broader range of walking speeds to better quantify the scope of accuracy in measurement for each device. Another limitation includes the dual-task effect of having the participants follow a researcher during FWS and SWS conditions. We recognize that based on this, the current values for 6MW are not comparable to those from studies that use a standard 6MW protocol [13]. As such, the reported values for 6MW distance, especially under FWS and SWS conditions, should be interpreted with caution, though this was not a primary outcome of the current study. There was a high proportion of persons with relapsing-remitting MS, despite the stratification of participants into EDSS groups of mild, moderate, and severe disability. A final limitation is that we did not measure the accuracy of each motion sensor during running. We were not interested in this modality of physical activity, based on the large range of MS disability, although it would be of interest to researchers examining the full extent of accuracy in measurement of the StepWatch activity monitor and the ActiGraph model GT3X+.

## Conclusions

Overall, we report that the ActiGraph model GT3X+ and StepWatch activity monitor both demonstrate highly accurate measurement of steps taken during comfortable and faster over-ground walking. This supports the inclusion of both motion sensors in clinical applications measuring steps/day. However, future researchers should be aware of a slight discrepancy in accuracy at slower walking speeds, particularly among those with severe disability, such that the ActiGraph model GT3X+ might underestimate steps taken by upwards of 10%. We believe that this is an intermediary between treadmill and real-world conditions that can help inform the choice on adopting accelerometers in clinical trials of MS wherein the monitoring of free-living walking behavior is of particular value.
